# Prediction of post-stroke depression with combined blood biomarkers IL-6, TNF-a, and fatty acid binding protein: A prospective study

**DOI:** 10.5937/jomb0-43904

**Published:** 2023-10-27

**Authors:** Linlin Wang, Chen Chunyou, Jingang Zhu, Xianjun Bao, Xiaoxiao Tao

**Affiliations:** 1 The First People's Hospital of Wenling, Department of Neurology, Zhejiang, China

**Keywords:** post-stroke depression, intestinal fatty acid binding protein, IL-6, TNF-a, depresija nakon moždanog udara, protein koji vezuje crevne masne kiseline, IL-6, TNF-a

## Abstract

**Background:**

To investigate the expression levels of blood biomarkers interleukin-6 (IL-6), tumour necrosis factor (TNF-a), and intestinal fatty acid binding protein (iFABP) in patients with post-stroke depression (PSD), and their correlation with PSD occurrence.

**Methods:**

Clinical data of stroke patients admitted to the First People's Hospital of Wenling from December 2017 to December 2022 were retrospectively analyzed. Patients were classified into two groups based on their Hamilton Depression Rating Scale (HAMD) scores: PSD and nonPSD groups. The blood levels of IL-6, TNF-a, and iFABP were compared between the two groups, and their association with PSD occurrence was analyzed.

**Results:**

The PSD group had significantly higher levels of IL-6, TNF-a, and iFABP. The combined detection of these biomarkers demonstrated a greater predictive value for PSD occurrence compared to the individual detection of each biomarker.

**Conclusions:**

The study indicates that the levels of IL-6, TNF-a, and iFABP in the blood are significantly increased in patients with PSD. The combined detection of these biomarkers can effectively predict the occurrence of PSD, indicating high clinical value.

## Abstract (sr)

**Uvod:**

Cilj je bio da se istraži ekspresija biomarkera u krvi interleukina-6 (IL-6), faktora tumorske nekroze (TNF-a) i proteina koji vezuje masne kiseline creva (iFABP) kod pacijenata sa depresijom nakon moždanog udara (PSD) i njihovim korelacija sa pojavom PSD-a.

**Metode:**

Retrospektivno su analizirani klinički podaci pacijenata sa moždanim udarom koji su primljeni u Prvu narodnu bolnicu Venling od decembra 2017. do decembra 2022. godine. Pacijenti su klasifikovani u dve grupe na osnovu rezultata Hamiltonove skale za procenu depresije (HAMD): PSD i ne-PSD grupe. Nivoi IL-6, TNF-a i iFABP u krvi su upoređeni između dve grupe i analizirana je njihova povezanost sa pojavom PSD.

**Rezultati:**

PSD grupa je imala značajno više nivoe IL-6, TNFa i iFABP Kombinovana detekcija ovih biomarkera pokazala je veću prediktivnu vrednost za pojavu PSD-a u poređenju sa pojedinačnom detekcijom svakog biomarkera.

**Zaključak:**

Studija ukazuje da su nivoi IL-6, TNF-a i iFABP u krvi značajno povećani kod pacijenata sa PSD. Kombinovana detekcija ovih biomarkera može efikasno predvideti pojavu PSD-a, što ukazuje na visoku kliničku vrednost.

## Introduction

Stroke is a leading cause of mortality and disease burden among people over 60 [1]. Around one in three of stroke survivors suffer from the painful of post-stroke depression (PSD), which is a prevalent psychological health condition with high mortality rate, low life of quality and severe dysfunction of cognitive [2] [3]. The symptoms of depression, including fatigue, may affect the assessment of recovery in stroke patients. Therefore, it is crucial to take proactive measures to prevent and accurately diagnose post-stroke depression (PSD) in these patients.

Although depression is highly prevalent among stroke patients, few biological mechanisms have been identified as strongly correlated with its development. Several biological and psychological factors have been shown to play important roles in the development of PSD. The »gut leak« hypothesis proposes that stroke can alter gut permeability, leading to changes in gut microbiota that potentially produce various types of psychiatric symptoms through immune activation [4] [5]. Zhong et al. [6] have investigated that intestinal fatty acid-binding protein (iFABP) is a biomarker that indicates gut permeability and can detect gut leak. The iFABP could disturb the lipid metabolism [6]. Despite the established link between iFABP and lipid metabolism with PSD, there are few studies related to their association with the central nervous system.

Stroke patients often have elevated levels of proinflammatory cytokines, which can exacerbate the inflammatory process in specific areas of the brain. These accumulations can result in psychological dysfunction and even PSD [7]. Previous research has shown that elevated levels of interleukin-6 (IL-6) and tumour necrosis factor-α (TNF-α) are associated with the development of PSD [8] [9] [10] [11] [12], however, their ability to predict PSD has not been fully established.

IL-6 and TNF-α are markers of immune activation and have been implicated in signalling immune reactions during the PSD process. These cytokines may also interact with iFABP, potentially leading to gut permeability and subsequent immune and inflammatory responses [13] [14]. David et al. [15] demonstrated that patients with spinal cord injuries have elevated levels of IL-6, TNF-α, and iFABP, which are associated with increased gut barrier damage. We propose that elevated levels of IL-6, TNF-α, and i-FABP may improve the predictability of PSD. Currently, there is a lack of effective method to predict the onset of PSD, emphasizing the imperative for further research in PSD populations. Therefore, the aim of this study is to assess the predictive value of combining iFABP, IL-6, and TNF-α testing for the onset of PSD in hospitalized stroke patients.

## Materials and methods

### Clinical data

Our study was carried out with the approval of the Clinical Research Ethics Committee (KY-2021-1047-01), From December 2017 to December 2022 in the First People's Hospital of Wenling, 91 patients with post-stroke depression (PSD group) and 208 patients who did not exhibit depressive symptoms after stroke (stroke group) were recruited into this study. Inclusion criteria were as follows: (1) Diagnosis of ischemic or haemorrhagic stroke with age 18 years; (2) Completion of relevant imaging (computed tomography (CT) or magnetic resonance imaging (MRI)) and depression scale examinations; (3) Firstonset stroke; (4) No use of antidepressant medication before admission; (5) Signed informed consent to use blood samples and related information for medical research. Exclusion criteria were as follows: (1) Patients who were unable to complete relevant examinations (including imaging and depression scale tests); (2) Patients with a history of other mental illness or relevant family history before stroke; (3) Patients with organ failure such as heart, liver, or kidney; (4) Patients with incomplete clinical data due to self-withdrawal, transfer to another hospital, death, and other reasons. Patient information such as age, gender, smoking history, drinking history, history of hypertension, history of diabetes, etc., was collected at the time of admission.

### Scales for stroke and depression

Within one day of admission, patients' neurological function deficits were assessed using the National Institutes of Health Stroke Scale (NIHSS). The NIHSS is an impairment scale with 15 items to evaluate the severity of stroke [16]. Patients were asked to complete the scale either independently or with assistance from our researchers or their families.

After discharge, patients were followed up and assessed for their depression levels using the 24-item Hamilton Depression Scale (HAMD) [17]. The HAMD is one of the most commonly used scales for assessing the severity of depression. Patients who score between 0 and 7 are considered to have no depression, whereas those who score 8 or above are categorized as having mild or more severe depression and are included in the PSD group.

### Blood test of IL6, TNF-α and iFABP

Blood samples were collected from patients using ethylenediaminetetraacetic acid (EDTA) anticoagulant immediately after admission and before treatment. Fasting plasma was obtained by centrifuging at 4000 rpm for 5 minutes and stored at -80°C. The concentrations of intestinal fatty acid-binding protein (iFABP), interleukin-6 (IL-6), and tumour necrosis factor-alpha (TNF-α) were measured using a commercially available enzyme-linked immunosorbent assay (ELISA) kit and sandwich ELISA method with specific antibodies. The ELISA kits used were E-EL-H0159c (Elabscience, United States) for iFABP, E-EL-H0109c (Elabscience, United States) for IL-6, and Abcam (ab178013) for TNF-α.

### Statistical analysis

Statistical analysis was performed using Statistical Product and Service Solutions (SPSS) 23.0 software (IBM, Armonk, NY, USA). Count data were presented as frequencies, and the chi-square test was used for testing significance. Continuous data were expressed as mean ± SD, and group comparisons were conducted using the t-test. Orthogonal projections to latent structures (OPLS) was used to establish a relationship model between metabolite expression and sample categories in R language, allowing for sample classification. Pearson correlation analysis examined the correlation between iFABP, IL-6, TNF-α, and NIHSS scores, while logistic regression analysis analyzed the relationship between iFABP, IL-6, TNF-α, and PSD occurrence. ROC curves were utilized to analyze the predictive value of the three observation indicators combined for PSD occurrence. P<0.05 indicated a statistically significant difference.

## Results

### Comparison of general characteristics

A total of 91 post-stroke depression (PSD) cases and 208 post-stroke non-depression cases were included in this study. There were no significant differences observed between the two groups in terms of age, gender, smoke, alcohol, hypertension, diabetes, type of stroke, and treatment methods. However, the NIHSS and HAMD scores in the PSD group were significantly higher than those in the stroke group (Table 1).

**Table 1:** Characteristics of patients in PSD and stroke group.

Category	PSD<br>(n=91)	Stroke<br>(n=208)	t/χ^2^	P
Age	65.6±15.3	63.1±14.3	1.48	0.14
Sex (M/F)	63/28	147/61	0.06	0.80
Smoke (Yes/No)	31/60	72/136	0.01	0.97
Alcohol (Yes/No)	30/61	91/117	3.06	0.08
Hypertension (Yes/No)	69/22	141/67	1.96	0.16
Diabetes (Yes/No)	33/58	72/136	0.08	0.78
Stroke (Ischemic/hemorrhagic)	75/16	157/51	1.75	0.19
Drugs<br>Antiplatelet (Yes/No)<br>Anticoagulant (Yes/No)	<br>51/40<br>23/68	<br>106/102<br>43/165	<br>0.78<br>0.66	<br>0.42<br>0.38
HAMD	14.1±5.5	3.0±1.7	23.56	<0.001
NIHSS	11.1±4.6	9.9±4.9	2.20	0.03

### Establishment and Evaluation of the OPLS Model

**Figure 1:**
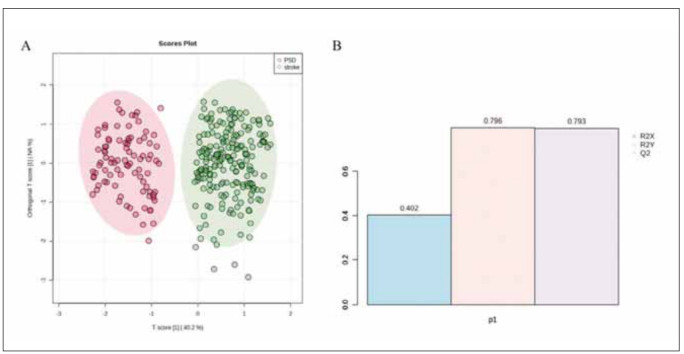
OPLS model established for PSD and stroke groups: (A) Score plot of the OPLS model (stroke patients: green dots; PSD group: red dots); (B) Overview of the model.

We used the measured values of iFABP, IL-6, and TNF-α in both PSD and non-PSD stroke patients to construct an OPLS model and evaluate the relationship between the expression levels of these biomarkers and group classification. As demonstrated in Figure 1, PSD patients and non-PSD stroke patients were distinctly separated, indicating significant differences between the two groups. The model's R2Y and Q2 values suggested good interpretability and predictability, confirming its effectiveness.

### Correlation between iFABP, IL-6, TNF-α and PSD

T-tests were performed to investigate the correlation between iFABP, IL-6, TNF-α levels and the occurrence of PSD. Our findings demonstrate that the levels of iFABP, IL-6, and TNF-α were significantly higher in the PSD group compared to the stroke group without PSD, as presented in Table 2.

**Table 2:** Levels of iFABP, IL-6, and TNF-α in PSD and stroke groups. *P<0.05

Biomarker	PSD	Stroke	t	P
iFABP<br>(μg/mL)	5.60±2.25	4.51±1.78	4.88	<0.001*
IL-6<br>(pg/L)	25.16±16.81	17.99±12.27	3.89	0.004
TNF-α<br>(pg/L)	30.74±17.87	25.45±13.11	2.93	<0.001*

In addition, Pearson correlation coefficients were further used to explore the correlation between iFABP, IL-6, TNF-α levels and NIHSS. The analysis revealed that iFABP and TNF-α levels were positively correlated with the severity of neurological damage, as demonstrated in Table 3.

**Table 3:** Correlation of the iFABP, IL-6, and TNF-α levels and NIHSS. *P<0.05

Biomarker	r	P
iFABP (μg/mL)	0.34	0.003*
IL-6 (pg/L)	0.16	0.19
TNF-α (pg/L)	0.26	0.03*

### PSD occurrence prediction with combined information of iFABP, IL-6, and TNF-α

Logistic regression analysis was used to explore the relationship between iFABP, IL-6, TNF-α, and the occurrence of PSD, and a predictive model was established. The results are shown in Table 4.

**Table 4:** Logistic regression results for combined iFABP, IL-6, and TNF-α levels.

Biomarker	B	S.E	Wald	P	OR	95%CI
iFABP<br>(μg/mL)	0.24	0.07	12.90	<0.001	1.27	1.11–1.44
IL-6<br>(pg/L)	0.03	0.01	9.34	0.002	1.03	1.01–1.05
TNF-α<br>(pg/L)	0.02	0.01	5.65	0.02	1.02	1.00–1.04

**Figure 2:**
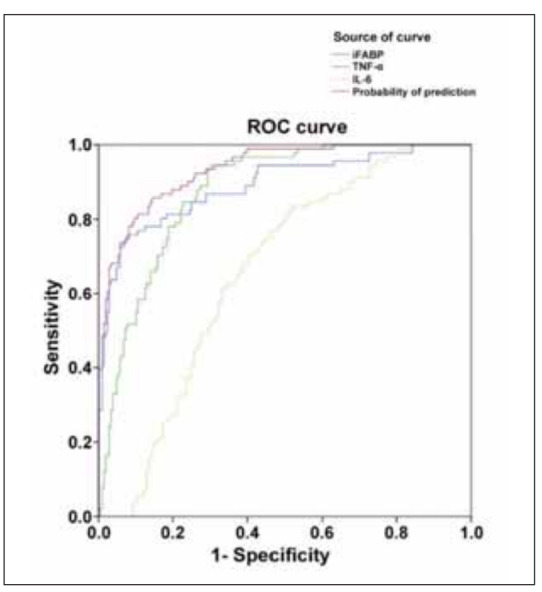
The ROC results of separate iFABP, IL-6, and TNF-α levels and combined these biomarkers levels for PSD prediction

Using this model, we calculated the probability of post-stroke depression (PSD) for each patient based on their measured levels of iFABP, IL-6, and TNF-α. We then constructed ROC curves using the probability of each patient to evaluate the predictive accuracy of combined iFABP, IL-6, and TNF-α and separate biomarkers levels. The results showed that iFABP alone had a higher predictive value than IL-6 and TNF-α alone. The AUC for the combined detection of iFABP, IL-6, and TNF-α was 0.933, which was higher than the AUC for each individual indicator (iFABP: 0.891; TNF-α: 0.874; IL-6: 0.658), as shown in Figure 2. These results suggest that PSD patients exhibit increased intestinal permeability. This discriminative model can effectively differentiate between PSD and stroke patients who do not experience depression.

## Discussion

PSD is a frequently occurring consequence among stroke patients, which significantly impairs their ability to cognitive function, quality of life, and even survival [2]. Nevertheless, the existing literature on the mechanisms, prevention, screening, diagnosis, and treatment of PSD is limited. Further clinical data is necessary to provide robust support in this area [18].

The »leaky gut hypothesis« has emerged as a significant explanation for the development of poststroke depression (PSD) in recent years. The iFABP is expressed exclusively in the intestinal epithelial cells of the small intestine and represents a promising biomarker for monitoring changes in gut permeability and integrity [19]. It has been shown to indicate early gut damage of intestinal barrier [20]. Several clinical studies have reported that microbial infiltrationinduced damage to the intestinal barrier is associated with defective schizophrenia, suggesting that barrier disruption may serve as a potential biomarker for disease onset and progression [21] [22] [23]. Stroke-induced changes in the gastrointestinal system, such as intestinal leakage, can cause dysbiosis and impair the braingut axis signal. This can hinder functional recovery after neurological damage and increase the risk of PSD [4].

Although the direct causal relationship between gut permeability and depression has not yet been fully understood, there is some research exploring the role of iFABP in the development of PSD [6] [24] [25] [26]. Clinical studies indicate that there is a correlation between the severity of depression and the increase in plasma iFABP levels among patients with PSD. Specifically, higher levels of iFABP in the plasma are associated with more severe depression symptoms [27] [28] [29]. Zhong et al. [6] found that PSD patients had significantly higher plasma levels of iFABP than stroke patients, and that these levels were positively correlated with NIHSS scores. This suggests that iFABP is linked to post-stroke depression and can serve as an indicator of the severity of neurological damage resulting from stroke. While their study only enrolled 48 post stroke patients.

IL-6 and TNF-α are two classical pro-inflammatory cytokines [30]. After a stroke, changes in gut permeability can allow the transportation of lipopolysaccharide (LPS) from the gram-negative bacterial cell wall into the bloodstream. This can activate immune cells and cause the secretion of pro-inflammatory cytokines, including IL-6 and TNF-α. These inflammatory factors can then interfere with neurotransmitters, disrupt synaptic activity of neurons, and increase the risk of developing depression [31] [32] [33]. Inflammatory factors may also produce cytotoxic damage to nerve cells by affecting the peripheral systemhypothalamus-pituitary-adrenal axis [34] [35] [36]. In our study, PSD patients have elevated levels of both IL-6 and TNF-α in their plasma. This study shows that these cytokines are also associated with the development of PSD, showing a moderate correlation with the severity of neurological damage.

## Conclusion

The results of this study indicate that the levels of plasma iFABP, IL-6, and TNF-α in the PSD group are significantly higher compared to that in the stroke group. This result suggests that the levels of iFABP, IL-6, and TNF-α are correlated with the occurrence of PSD and provide some support for the hypothesis of intestinal leakage in PSD. Further statistical analysis showed that the levels of iFABP, IL-6, and TNF-α were positively correlated with the occurrence of PSD, and the combined detection of iFABP, IL-6, and TNF-α had a higher predictive value for PSD than each biomarker alone. In summary, the combined detection of iFABP, IL-6, and TNF-α can effectively predict the occurrence of PSD, help us to further explore the basic principles of PSD occurrence, provide new support for the intestinal leakage hypothesis, and facilitate the screening of meaningful biomarkers, providing clues and evidence for clinical work.

## Dodatak

### Funding

This research was supported by the social development science and technology foundation of Wenling (2021S00145).

### Informed Consent Statement

The informed consent was obtained for all patients in this study. Written informed consent was obtained from the patients to publish this paper.

### Data Availability Statement

For patients' privacy protection, the data availability was not applicable.

### Conflict of interest statement

All the authors declare that they have no conflict of interest in this work.
